# Editorial: Inspired by Nature: Towards Novel Anti-Infective Agents

**DOI:** 10.3389/fphar.2022.843966

**Published:** 2022-01-21

**Authors:** Ricardo J. Ferreira, Mariana A. Reis, Daniel Silva, Elisandra Scapin, Saikat Dewanjee, Marta P. Carrasco

**Affiliations:** ^1^ Red Glead Discovery AB, Lund, Sweden; ^2^ Interdisciplinary Center for Marine and Environmental Research, University of Porto, Matosinhos, Portugal; ^3^ Unilever, London, United Kingdom; ^4^ Federal University of Tocantins, Palmas, Brazil; ^5^ Advanced Pharmacognosy Research Laboratory, Department of Pharmaceutical Technology, Jadavpur University, Kolkata, India; ^6^ Medicinal Chemistry, Faculty of Pharmacy, University of Lisbon, Lisbon, Portugal

**Keywords:** antibiotics, antivirals agents, drug development, eutectic liquids, natural products

Since the dawn of mankind natural sources have been explored due their valuable medicinal properties. Even in modern times several compounds are still being identified in the natural source(s) from which anti-microbial activity was originally isolated. However, the world of natural products is still the largest unexplored chemical space when compared with the largest of the known chemical databases. Although remaining one of the best sources of chemical inspiration for the development of new drugs, only around a quarter million natural products are available within public databases and no more that ten percent can be readily obtainable from commercial vendors for experimental testing. The identification of such molecular scaffolds, either derived from plants, microorganisms or marine products, followed by isolation of active molecules and further chemical modification, allows researchers to further improve their pharmacological properties. Having strong roots in traditional medicine, in which plants and plant extracts only known to the medicine caregivers are used to treat all sorts of diseases, the identification of each compound responsible for that given activity is usually the first step in increasing the pool of chemical templates. This is why many compounds were first isolated from natural sources and used to develop drugs targeting specific diseases, from which salicylic acid, morphine and quinine are classical examples. However, due to the intricate complexity of some molecules, hemi-synthetic derivatives as etoposide and teniposide, paclitaxel or vinblastine/vincristine remains as the most feasible approach for obtaining more potent compounds in selected instances.

The main challenge within this topic was to use nature as an inspiration for new compounds that can pave the way for future pharmacological innovations. In this perspective, boron-containing DNA-binding ionic liquids as antimicrobial agents (Rosa et al.) or inspired by natural product nitrogen-containing 5-membered heterocyclic moieties (Camargo et al.) are two very interesting approaches in the development of new antibiotics. The identification of sugar-type compounds as antiviral agents is also remarkable (Zhou et al.), taking into account that generally sugar moieties are often ignored in drug development. Furthermore, novel techniques such as Molecular Dynamics, largely ignored in the natural products, are slowly increasing in importance as useful tools for the comprehension of more dynamic systems as nature-inspired eutectic solvents (Monteiro et al.).

Taken together these papers present a glimpse on the opportunities that nature continues to provide, when properly addressed. Therefore, further inspiration can be obtained by harnessing the enormous potential delivered by nature throughout its natural selection and evolution.

